# Comparison of fluoroscopy time and procedure time of endovascular interventions with and without prior angiography simulator training: a meta-analysis

**DOI:** 10.1186/s41077-025-00382-y

**Published:** 2025-10-27

**Authors:** Timo C. Meine, Johannes M. Dorl, Anselm A. Derda, Nima Mahmoudi, Hans-Jonas Meyer

**Affiliations:** 1 Institute for Diagnostic and Interventional Radiology, Hannover Medical School, Carl-Neuberg-Strasse 1, 30625 Hannover, Germany; 2 PRACTIS Clinician Scientist Program, Dean’s Office for Academic Career Development, Hannover Medical School, Carl-Neuberg-Strasse 1, 30625 Hannover, Germany; 3 Hannover Medical School, Carl-Neuberg-Strasse 1, 30625 Hannover, Germany; 4 Department of Cardiology and Angiology, Hannover Medical School, Carl-Neuberg-Strasse 1, 30625 Hannover, Germany; 5 Institute of Molecular and Translational Therapeutic Strategies, Hannover Medical School, Carl-Neuberg-Strasse 1, 30625 Hannover, Germany; 6 Institute of Neuroradiology, Charite–Universitätsmedizin Berlin, Chariteplatz 1, D–10117 Berlin, Germany; 7 Institute for Diagnostic and Interventional Neuroradiology, Hannover Medical School, Carl-Neuberg-Strasse 1, 30625 Hannover, Germany; 8 Department of Diagnostic and Interventional Radiology, University Hospital Leipzig, Liebigstrasse 18, 04103 Leizpig, Germany

**Keywords:** Meta-analysis, Simulator, Training, Angiography, Endovascular, Intervention

## Abstract

**Background:**

Angiography simulator training (AST) can help to train important clinical aspects of complex angiography procedures before real patient contact. The aim of the present analysis was to synthesize the results of studies on endovascular interventions performed by interventionalists with and without AST in a meta-analysis.

**Methods:**

A systematic literature research was performed in PubMed, Web-of-Science and CINAHL to identify all relevant studies. Inclusion criteria were original research, English language and comparison of endovascular interventions in procedure time (PT) and fluoroscopy time (FT) performed by interventionalists with and without AST. Study quality was assessed using modified Downs-and-Black-instrument (maximum 8 points). Heterogeneity-analysis (study design and I^2^) was determined, and fixed- or random-effects model was applied to pool the effect, mean difference (MD), from the individual studies. All analyses were performed two-sided, and the level-of-significance was 0.05.

**Results:**

Overall, 9 studies with 10 datasets and 7774 interventions were included. Study quality was 7 ± 0 for both PT and FT. Heterogeneity was present in the studies on PT (*I*^2^ = 61%) and FT (*I*^2^ = 99%), and a random-effects model was applied. MD for PT was significant with −2.63 min between the AST-group and control-group among the included studies (*p* = 0.02). In contrast, MD was not significant with −1.33 min between the AST-group and control-group among the included studies for FT (*p* = 0.21).

**Conclusion:**

AST translates into an improved PT and similar FT in real interventions compared to conventional training. Angiography simulators offer a valuable, radiation-free alternative and expand training opportunities. Evidence is limited by study heterogeneity.

**Supplementary Information:**

The online version contains supplementary material available at 10.1186/s41077-025-00382-y.

## Background

Indications for minimally invasive therapies, especially angiographic or endovascular interventions, are increasing, whereas indications for open surgery are decreasing in a society with a growing number of elderly patients [1, 2]. The ever-growing demand for endovascular procedures goes hand in hand with a growing need for endovascular specialists.

However, opportunities for training in endovascular skills have decreased since diagnostic angiographies with lower skill requirements are often replaced by cross-sectional imaging nowadays [3].

Thus, education in endovascular procedures is important to maintain safe and efficient treatment for the growing number of patients.

Therefore, simulation training of interventions is a common practice in various health systems. Classical phantom models of humans or patients’ vascular anatomy, e.g. silicon models, have been used for years with promising results [4].

In recent years, electronic simulators for training of endovascular interventions have been developed, similar to flight plane simulators that have improved pilot training [5]. Several studies have investigated the efficacy of angiography simulator training (AST) for real patient interventions using common efficacy parameters, procedure time (PT), and fluoroscopy time (FT) as clinically relevant outcome parameters [6–8].

However, to the best of our knowledge, no systematic overall evaluation with quantitative synthesis of the benefit of endovascular or angiography simulators for the efficacy of real interventions has been conducted. This is especially important as the costs of the simulator models and time demand for the training can be high.

Thus, the present analysis aims to synthesize FT and PT results of studies on endovascular interventions performed by interventionalists with and without prior AST in a meta-analysis.

## Material and methods

### Systematic literature research

The electronic literature databases PubMed, Web of Science and CINAHL were searched by applying an advanced search in the category “all fields” with the following terms: ((*angiography simulator*) *OR* (*endovascular simulator*)) *AND* ((*fluoroscopy time*) *OR* (*procedure time*)). No restriction of the time interval or language was selected. Literature research was finally performed on 17 January 2025. Database-registration was not performed due to indirect impact on health outcomes [9]. Detailed search terms were given in Supplements.

### Study inclusion

Inclusion criteria for the meta-analysis were original research, English language and comparison of endovascular interventions in PT and FT performed by interventionalists with and without AST (AST-group and control-group). Endovascular or angiography simulator, PT and FT were defined as follows:

Endovascular or angiography simulators were defined as high-fidelity electronic simulators. These electronic simulators are virtual reality systems with at least one haptic device box and one computer with a control panel and display [10]. In the haptic device box, interventional devices (guidewires, catheters etc.) can be inserted and syringes can be attached. C-arm and table position, fluoroscopic series, digital subtraction angiography series and more aspects of a real angiography can be simulated [10]. In addition, it is possible to upload computed tomography images on the electronic simulator [10]. After training on the simulator, this intervention can be performed on a real patient (specific rehearsal) [10].

PT was defined as the time required for the whole intervention.

FT was defined as the time exposed to radiation during the intervention.

Exclusion criteria were single-arm studies, no comparison of groups with and without angiography simulator training, different outcome parameters, and no comparison of real interventions in patients.

Screening of the literature for study inclusion was performed independently by two investigators (J.M.D. and T.C.M.) in a standardized format according to the Preferred Reporting Items for Systematic Reviews and Meta-Analysis (PRISMA) statement [11, 12]. Disagreements were resolved in discussion and a group consensus was reached. No other studies could be identified via citation analysis of identified studies. Reference manager Zotero (https://www.zotero.org/) was applied.

### Data extraction

Data on the first author, year of publication, country, design, interventionalists, intervention, simulator, training type, procedure success, (procedure-related) complication rate, radiation exposure (dose area product) and defined outcome parameter (PT and FT) were extracted from the included studies. For the outcome parameter, number of interventions, as well as mean and standard deviation, were extracted. Two investigators (J.M.D. and T.C.M.) extracted the data independently in a standardized format. When disagreements occurred, a group consensus was reached. Different reported values, e.g. median and interquartile range, were transformed in mean and standard deviation as recommended in the Cochrane Handbook [13–15] or published in the literature [16–20]. Details were given in Supplements.

### Study quality assessment

Study quality was analysed using a modified Downs-and-Black instrument, as published by Zadro et al. [21]. The maximum score of this modified version was 8 points. Two investigators (J.M.D. and T.C.M.) independently analysed the quality of the included studies and a consensus was reached in case of disagreements. Values were given as mean and standard deviation.

### Statistical meta-analysis

First, heterogeneity was assessed by analysing the study design, using a visual approach with forest plots, and calculating the I^2^ of the included studies. Then, a fixed- or random-effects model was applied to combine outcome effects and mean difference (MD) between the AST- and the control-group. Publication bias analysis was conducted using funnel plots and Egger’s test. Subgroup analysis was performed according training type, intervention type, experience of the interventionalists, study size and simulator type. Finally, leave-one-out sensitivity analyses were conducted to explore the impact of each dataset on the pooled effect. *p*-values in this study were two-sided, and the alpha level was set at 0.05 for statistical significance. All analyses were performed with SPSS (IBM SPSS Statistics, Version 29, New York, USA). MD, 95% Confidence Interval (CI), and *p*-values were given.

## Results

### Systematic literature research and study inclusion

**Fig. 1:**
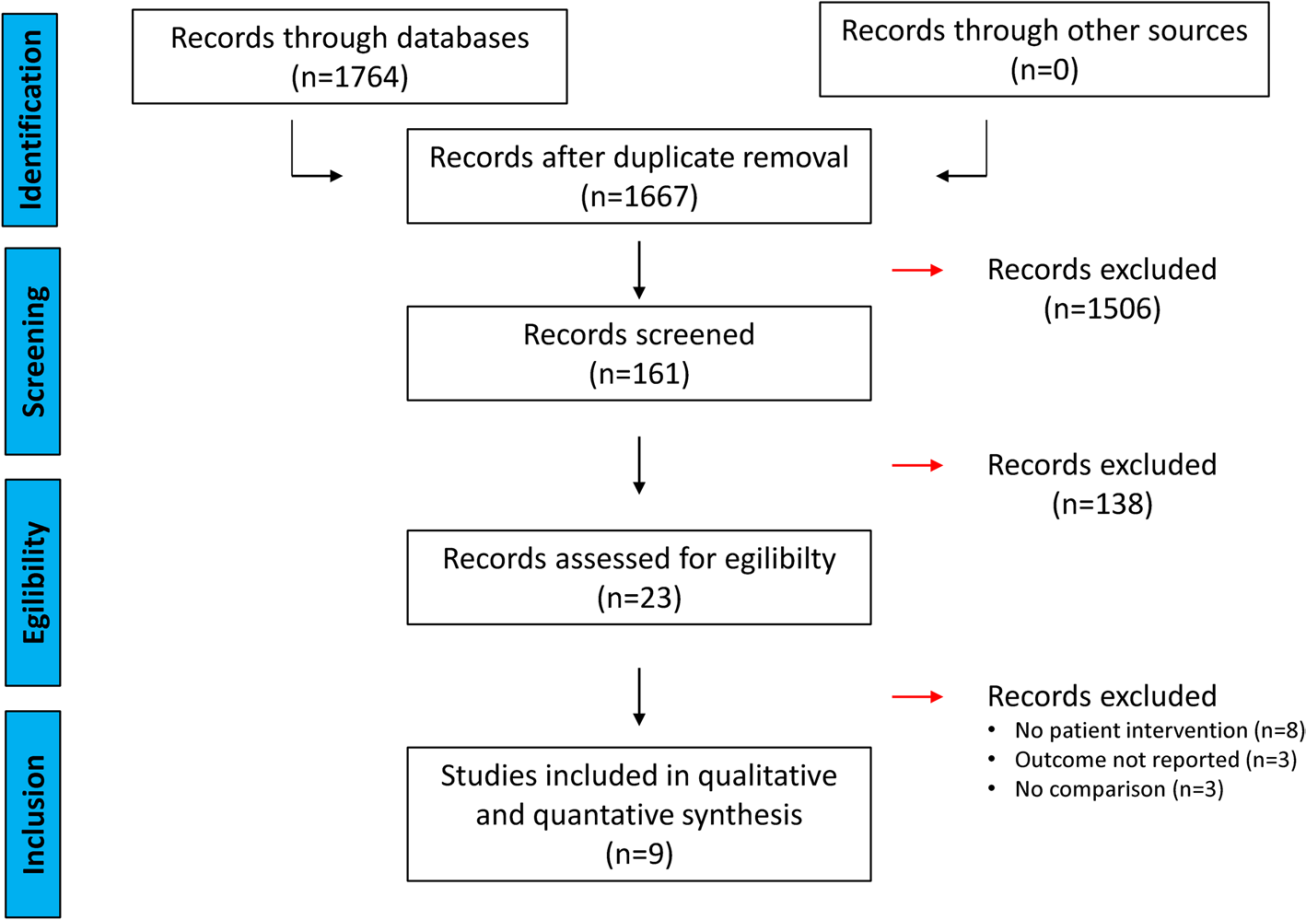
Overview of the literature screening in adaption to the PRISMA statement Abbreviations: *n* = number, PRISMA = Preferred Reporting Items for Systematic Reviews and Meta-Analysis

A total of 1764 records were collected from PubMed, Web of Science and CINAHL. Therefore, 97 doublets were removed. Screening the titles of the remaining 1667 records, we found 161 records of relevant topics. Then, abstracts were screened for study inclusion. Thereafter, 23 full texts were examined for study inclusion. Finally, 9 studies fulfilled the inclusion criteria and were included in the meta-analysis [6–8, 22–27]. Of note, one study by Kreiser et al. reported two different datasets without data overlap [24]. Thus, both datasets were separately included in this meta-analysis. In Fig. 1, the study in- and exclusion process is provided in adaption to the PRISMA statement.

### Data extraction

Overall, data of 7774 interventions, 10 datasets and 9 studies were extracted. The included studies were conducted in Sweden, Norway, Germany, France, Belgium and the USA and were published between 2014 and 2021. In five studies, the study design was randomized in selection of the cases or interventionalists. Three studies had retrospective study design and the study by Kreiser et al. reported one dataset with retrospective and one dataset with randomized design [24]. Further, the experience of the interventionalists varied among the studies. In five studies, physicians with interventional experience (e.g. consultant-level or consultant-level with interventional specification) performed the interventions. Physicians with limited experience (e.g. resident-level) performed the interventions in four studies, including five datasets. In addition, the physicians’ discipline was different. Physicians were affiliated with departments of surgery, cardiology and radiology. The electronic simulator used was the vascular interventional simulation trainer (VIST) (Mentice AB, Goeteborg, Sweden) in six studies (seven datasets) and the Angio Mentor (3D Systems Corporations, Rock Hill, SC/USA) in three studies. Simulated procedures were neurovascular angiography (NA) in three studies (four datasets), coronary angiography (CA) in four studies and endovascular aortic repair (EVAR) in two studies. Five studies reported AST with generic cases, whereas two studies reported patient- or procedure-specific rehearsal. In addition, the aforementioned study by Kreiser et al. reported training with generic cases in dataset A and specific rehearsal in dataset B [24]. Procedure success was only investigated in a single study with no significant difference between AST- and control-group. Complications were not reported in three studies and did not occur in another three studies. Considering major errors as procedure-related complications, there were less complications in the AST-group than in the control-group in the study by Desender et al. [8]. Complication rate at the catheter laboratory and number of diffusion weighted imaging lesions in the brain were not significant different in the studies with and without AST by Jensen et al. and Kreiser et al. [22, 24]. Radiation exposure, assessed with the dose area product, was not reported in three studies. In two studies, radiation exposure was lower in intervention performed by interventionalists with AST than without AST. In most studies (four studies and five datasets), there was no difference in the radiation exposure between AST- and control-group.

Finally, PT was reported in 8 studies (8 datasets) and FT in 9 studies (10 datasets). Overall, there were differences in study-design with varying interventionalist’s experience, different interventions, simulators and training. No significant differences between AST-group and control-group for PT and FT were observed in the majority of the studies. Details were given Table 1.

**Table 1:** Descriptive overview of the included studies

First author	Year	Country	Design	Interventionalist	Inter-vention	Simulator	Training	Procedure success	Complicat-ion rate	Radiation exposure^d^	Procedure time	Fluoroscopy time
Cates	2016	USA	Randomized	12 Interventional cardiologists	NA	VIST	Generic	n.a	n.a	n.a	AST = control	AST = control
Desender	2016	Belgium	Randomized	6 Centres	EVAR	Angio Mentor	Specific	n.a	AST < control’^c^	AST = control	AST = control	AST = control
Jensen	2014	Sweden	Retrospective	58 Novices	CA	VIST	Generic	n.a	AST = control”	n.a	n.a	AST > control^c^
Jensen	2016	Sweden	Randomized	16 Senior residents	CA	VIST	Generic	n.a	AST = control^a^	AST = control	AST < control^c^	AST < control^c^
Kreiser (A)	2020	Germany	Retrospective	6 Novices	NA	VIST	Generic	n.a	n.a	AST = control	n.a	AST < control^c^
Kreiser (B)	2020	Germany	Randomized	resident/senior physician°	NA	VIST	Specific	n.a	AST = control^b^	AST = control	AST = control	AST = control
Popovic	2019	France	Randomized	20 Residents	CA	Angio Mentor	Generic	n.a	AST = control^a^	AST < control^c^	AST < control^c^	AST = control
Prenner	2017	USA	Retrospective	12 Cardiologists	CA	VIST	Generic	n.a	n.a	AST < control^c^	AST < control^c^	AST = control
Våpenstad	2021	Norway	Retrospective	1 Surgeon and 1 radiologist	EVAR	VIST	Specific	AST = control	n.a	AST = control	AST < control^c^	AST = control
Wooster	2018	USA	Randomized	1 Centre	NA	Angio Mentor	Specific	n.a	AST = control^a^	n.a	AST = control	AST = control

*Abbreviations*: *AST* angiography simulator training, *CA *coronary angiography, *NA *neurovascular angiography, *n.a.* not available, *EVAR *endovascular aortic repair, *USA *United States of America, *VIST *vascular interventional simulation trainer. °Interventions were performed by residents in 25 cases and by senior physicians in 15 cases. ’Data on major errors were considered comparable to procedure-related complications. “Data on complications at the catheter laboratory were shown

^a^No procedure-related complication occurred

^b^Data on lesions on diffusion weighted imaging were shown

^c^Results in the reported literature were significant

^d^Results of reported dose area product were shown

### Study quality

The included studies had heterogenous designs, e.g. differences in the level of interventionalist’s experience and interventions and training types. Overall study quality was 7 ± 0 for both PT and FT in the Downs-and-Black instrument modified by Zadro et al., demonstrating good overall study quality [21]. Of note is that assessment of the appropriateness of statistical tests was not possible in all studies due to insufficient reporting. The results of the study quality assessment are given in Supplements.

## Statistical meta-analysis

### Procedure time

**Fig. 2:**
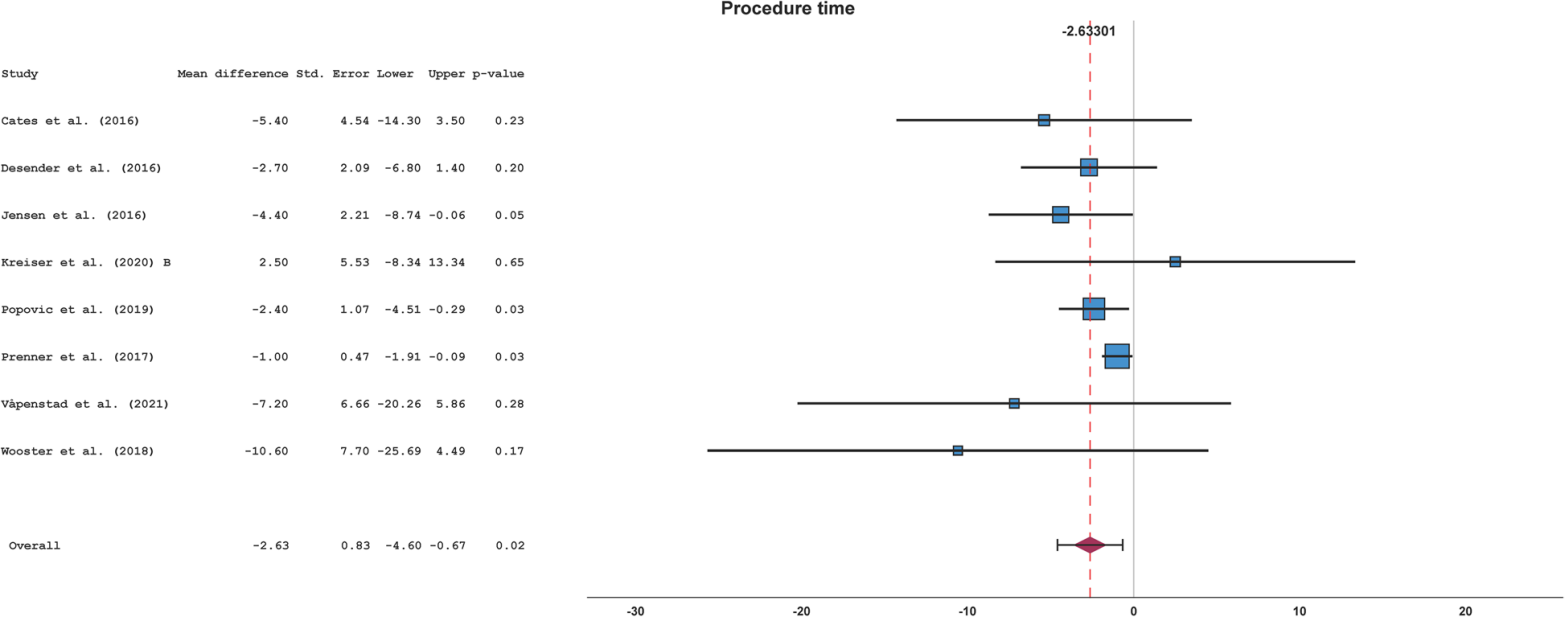
Forest plot of the procedure time Abbreviations: Std. Error = standard error, Upper = upper confidence interval border, Lower = lower confidence interval border

Of 9 included studies, 8 studies reported 8 datasets with PT of overall 3122 interventions. Heterogeneity was present among the studies with I^2^ of 61% and there were differences in interventionalists’ experience, interventions, simulators and training type among the published studies. Thus, a random-effects model, Sidik-Jonkman, with Knapp-Hartung error adjustment was applied. Overall MD for PT between AST- and control-group among the studies was −2.63 min (95% CI, −4.60 to −0.67 min; *p* = 0.02) (see Table 2 and Fig. 2).

**Table 2:** Procedure time

Study	AST-group		Control-group	
	*n*	Mean ± SD [min]	*n*	Mean ± SD [min]
Cates et al. (2016) [27]	6	26.9 ± 8.3	6	32.3 ± 7.4
Desender et al. (2016) [8]	50	57.0 ± 10.2	50	59.7 ± 10.7
Jensen et al. (2014) [22]	n.a	n.a	n.a	n.a
Jensen et al. (2016) [23]	16	22.9 ± 5.9	16	27.3 ± 6.6
Kreiser et al. (2020) A [24]	n.a	n.a	n.a	n.a
Kreiser et al. (2020) B [24]	20	32.5 ± 20.1	20	30.0 ± 14.4
Popovic et al. (2019) [25]	40	13.6 ± 6.1	40	16.0 ± 3.0
Prenner et al. (2018) [26]	895	23.9 ± 11.8	1888	24.9 ± 10.8
Våpenstad et al. (2021) [7]	30	60.0 ± 25.9	30	67.2 ± 25.7
Wooster et al. (2018) [6]	6	31.9 ± 15.7	9	42.5 ± 12.8

*Abbreviations AST *angiography simulator training, *min *minutes, *n *number, *n.a. * not available, *SD *standard deviation

**Fig. 3:**
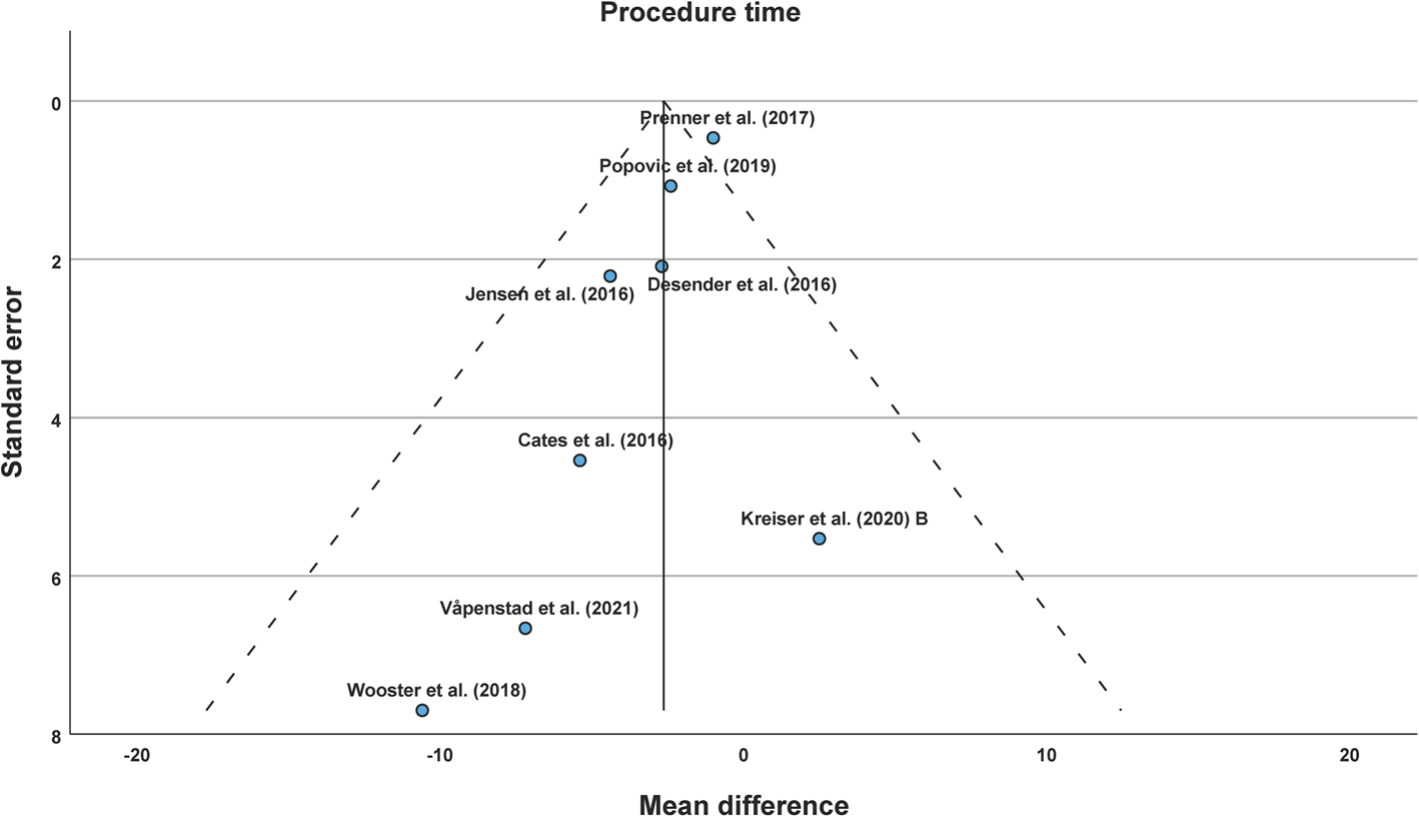
Funnel plot of the procedure time

No strong asymmetry in funnel plot was detected (see Fig. 3). Egger’s test was not significant (*p* = 0.26). Subgroup analyses on study design were significant with a MD of −3.16 min (*p* = 0.03) and −1.66 min (*p* = 0.54) for randomized and retrospective datasets, respectively (see Supplements). Other subgroup analyses were not significant (see Supplements). In leave-one-out sensitivity analyses, there was a borderline significance when Jensen et al. (2016) was left out. All other leave-one-out sensitivity analyses revealed significant results (see Supplements).

### Fluoroscopy time

**Fig. 4:**
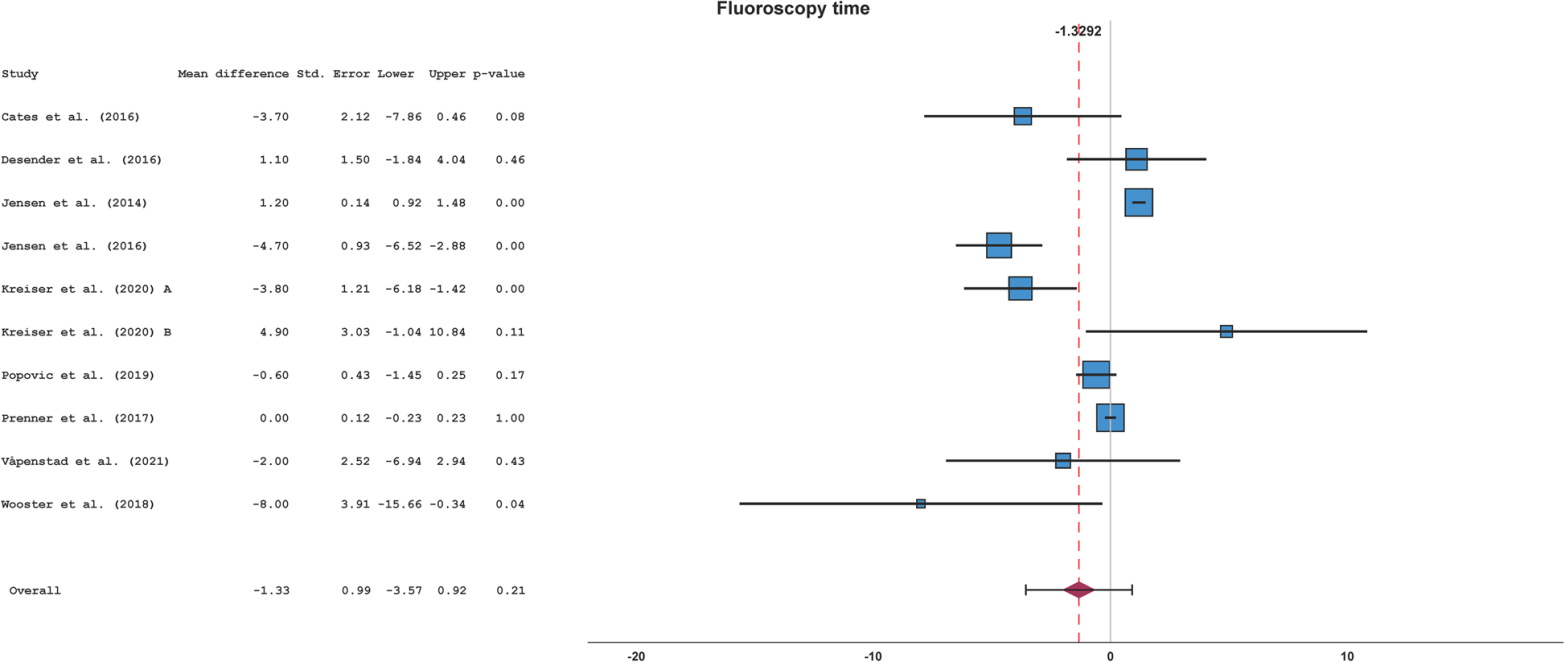
Forest plot of the fluoroscopy time Abbreviations: Std. Error = standard error, Upper = upper confidence interval border, Lower = lower confidence interval border

Overall, 9 studies reported 10 datasets with FT of 7774 interventions. In analogy to PT, the same differences in study design existed, and I^2^ was 99%. According to the present heterogeneity, the random-effects model Sidik-Jonkman with Knapp-Hartung error adjustment was performed for effect pooling. FT between AST- and control-group among the studies was not different with an overall MD of −1.33 min (95% CI, −3.57 to 0.92 min; *p* = 0.21) (see Table 3 and Fig. 4).

**Table 3:** Fluoroscopy time

Study	AST-group		Control-group	
	*n*	Mean ± SD [min]	*n*	Mean ± SD [min]
Cates et al. (2016) [27]	6	13.6 ± 2.6	6	17.3 ± 4.5
Desender et al. (2016) [8]	50	18.7 ± 7.7	50	17.6 ± 7.3
Jensen et al. (2014) [22]	878	6.4 ± 3.8	3594	5.2 ± 3.5
Jensen et al. (2016) [23]	16	9.2 ± 1.5	16	13.9 ± 3.4
Kreiser et al. (2020) A [24]	90	7.2 ± 8.8	90	11.0 ± 7.4
Kreiser et al. (2020) B [24]	20	13.0 ± 13.0	20	8.1 ± 3.8
Popovic et al. (2019) [25]	40	7.0 ± 1.5	40	7.6 ± 2.3
Prenner et al. (2018) [26]	895	4.9 ± 3.0	1888	4.9 ± 2.7
Våpenstad et al. (2021) [7]	30	28.7 ± 9.5	30	30.7 ± 10.0
Wooster et al. (2018) [6]	6	11.4 ± 5.6	9	19.4 ± 9.5

*Abbreviations*: *AST* angiography simulator training, min minutes, *n* number, *SD* standard deviation

**Fig. 5:**
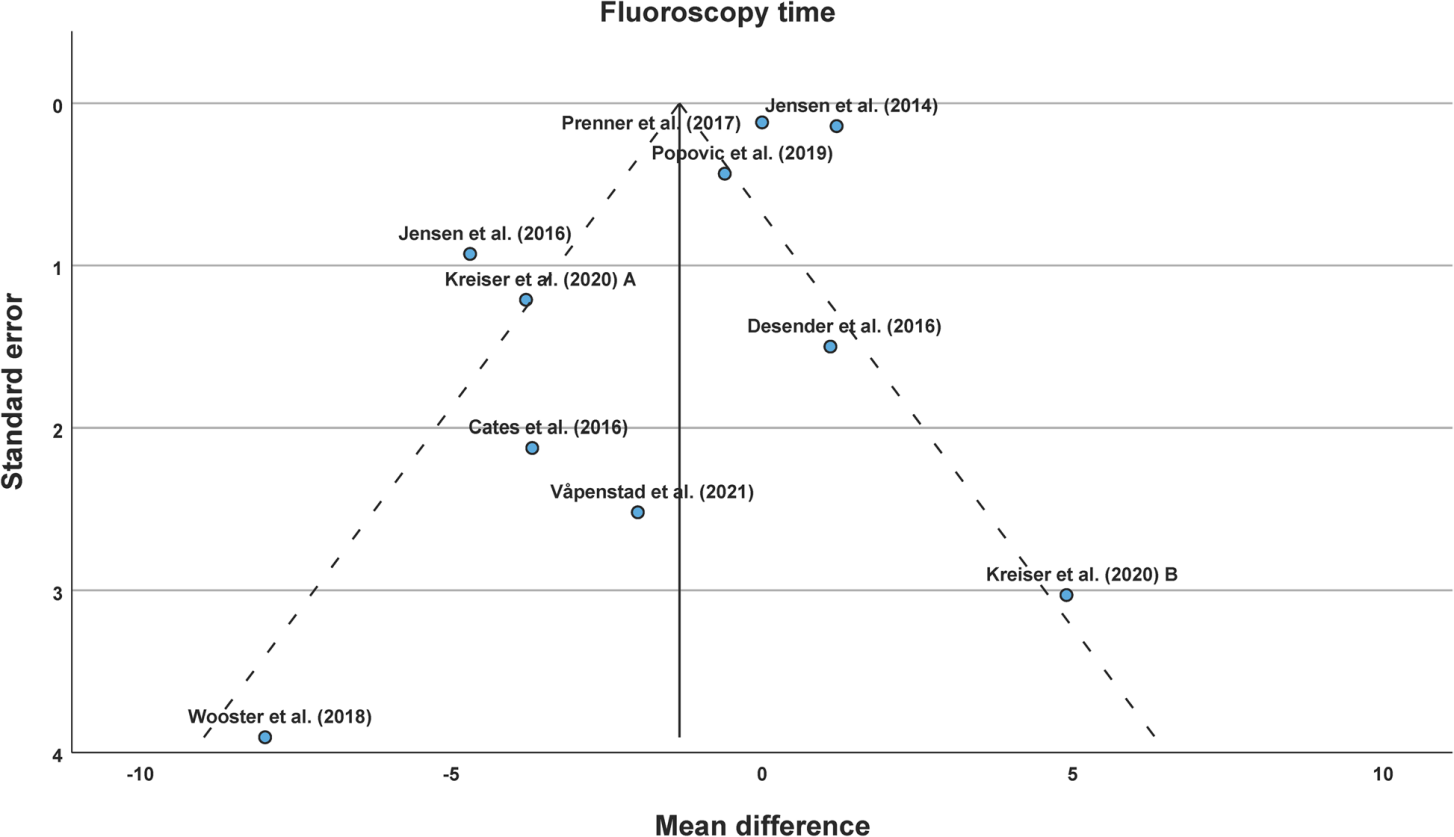
Funnel plot of the fluoroscopy time

There was no strong asymmetry in funnel plot (see Fig. 5). Egger’s test was not significant (*p* = 0.77). Subgroup analyses and leave-one-out sensitivity analyses for FT were not significant (see Supplements).

## Discussion

Reviewing our main findings, we have identified 9 studies and 10 datasets that compare FT and PT between groups with and without AST. In the quantitative synthesis, the pooled effect is significant with moderate reduction of the PT for the AST-group compared to the control-group. The effect on FT in real interventions is comparable between AST and conventional training. Both findings indicate the potential of radiation-free training capabilities with angiography simulators. This analysis provides, for the first, a quantitative synthesis of the benefit of simulator training before a real intervention with a patient is performed.

Focussed on the study characteristics, six of 9 studies have been conducted in Europe. Since both angiography simulators, VIST and Angio Mentor, have been invented and developed in Sweden, research in this field is expected to be conducted in European countries. Interestingly, the time of publication ranges between 2014 and 2021. Training on angiography simulators has already been incorporated in the curriculum for interventional training of certain medical societies, e.g. the Society of Cardiovascular Angiography and Interventions (SCAI) [28] and Cardiovascular and Interventional Society of Europe (CIRSE) [29]. This may explain why no further studies on the translation from simulator to patient intervention have been published in the last years, as it was already assumed to be beneficial.

Regarding the medical subspecialty, most endovascular procedures are conducted by cardiologists and (neuro-) radiologists. This may represent the main indications and applications for endovascular interventions, especially the many emergency interventions performed by cardiologists and (neuro-) radiologists.

Besides the countries and speciality, there are more study characteristics different. There was a randomized study design in six data sets and retrospective analysis in four datasets. Additionally, training type differed with five studies on generic training and four studies on specific rehearsal. Moreover, procedure success and complication rates were inconsistently reported. Procedure success was reported by Vapenstad et al. with no difference between AST- and control-group [7], which was also shown for EVAR by Desender et al. in separate publication [30]. Albeit one included study reported less major errors in AST-group compared to control-group [8], there were no different complication rates in most other studies. Radiation exposure was also reported with two studies favouring AST, but there was no difference in the majority of the studies. An overall conclusion on these data regarding AST cannot be drawn due to limited reporting. In analogy, reporting of statistical tests according the modified Downs-and-Black instrument was also insufficient which limits the overall fair study quality for both outcome parameters.

Considering the PT, the reported results of the literature (8 studies) are significant with reduced PT in AST-group in the half of the studies while there is no significance in the other half of the studies. Interestingly, the overall effect between AST- and control-group among all studies in our quantitative synthesis is significant with a MD of −2.63 min. This difference in PT is relatively small, but mean PT ranges from 16 to 67 min due to the different complexity of the interventions (see Tables 1 and 2). For example, EVAR procedures are associated with an increased intervention time, whereas coronary angiographies are performed in a comparably short intervention time, as reflected by the aforementioned PT values [7, 25]. Thus, the likelihood of reducing intervention time is higher for complex and time-intense interventions than for diagnostic angiographies. Although our subgroup analyses were not clearly different compared to the overall meta-analysis, a greater effect with specific training is conceivable. Training with the exact anatomy could benefit both interventionalists with limited experience and experienced interventionalists, especially in complex cases. Procedure steps of interventions as well as handling and connection of the devices can be trained with angiography simulators outside the patient. Although there is a large range of PT and a small number of studies on different interventions, the overall MD of all studies is still significant. This shows the clinical benefit of training procedural steps on an angiography simulator. It could lead to an improved time usage of the angio-suite and more patient comfort due to time reduction.

In review of the literature on FT (9 studies), FT was not different in six studies between the AST-group and the control-group. FT increased in the AST-group in one study and decreased in the AST-group in another. In the last study with two datasets, FT was reduced in AST-group in dataset A, whereas there was no difference in FT in dataset B. According to the literature, our meta-analysis finds no significant difference between AST and control-group among the studies with an overall MD of −1.33 min. Pooled effects are even smaller for FT than for PT. Thus, it is not unexpected that these results are not significant. An explanation could be the limited simulation of unexpected and uncontrollable motion of the patient (e.g. breathing) at the moment. When FT of real interventions is comparable between AST and conventional training, AST offers alternative, radiation-free and individual training opportunities for residents and interventionalists in training. This is relevant, since the number of angio-suites is limited and the number of diagnostic angiographies is decreasing as explained in the introduction. Of course, training general interventional techniques on angiography simulators (e.g. cross-over catheterization of aortic bifurcation or configuration of a sidewinder-catheter) and training spatial imagination ought to have an impact on FT in real patients. Experienced interventionalists may have sufficient device handling and spatial imagination, but might benefit from patient-/procedure-specific rehearsal for complex cases.

In subgroup analyses, MD was significant in PT for randomized datasets but not for retrospective datasets. This could indicate an effect of randomization for simulation training, but it has to be taken with caution due to the limited number of two retrospective studies in this subgroup. When Jensen et al. (2016) was left out in PT, results were borderline significant but still comparable to the overall analysis. Nevertheless, there is significant heterogeneity among the studies. Studies on CA have reported on novices or residents with AST on generic cases while most studies on NA and EVAR have reported on experienced interventionalists with specific rehearsal training. There are important differences between the interventions, e.g. the typical time for EVAR is expected to be twice the time for CA. In addition, catheterization and imaging of aortic branches or cerebrovascular arteries is different to the moving anatomy of coronary arteries. Although PT and FT can be monitored to assess the progress of interventionalists in training, different outcome parameters are more clinically important, e.g. endograft position and endoleak incidence after EVAR. However, the number of studies on AST with evaluation of training effects in subsequent intervention in real patients is still very limited.

There are several limitations in our study. First, it is a relatively small number of identified studies with varying sample sizes of primary data. Although no publication bias is indicated, the number of studies limit this analysis. Second, the available studies have differences in the study quality and design with different levels of interventionalist experience, interventions, simulators and training types. All these factors contribute to the heterogeneity among the studies and limit the evidence of the findings. Third, there has been neither standardized definitions nor standardized reporting of the simulation data in the studies, e.g. training time. This further underlines the heterogeneity of the included studies and limits the level of evidence.

Finally, future studies on simulator training could be improved to evaluate the training effect of angiography simulators. First, a standardized definition of training time on the angiography simulator, e.g. simulator hours, is important for objective reporting and assessment of training on angiography simulators. Second, the type of training should be also be standardized in adaption to the complexity of the intervention and the level of experience of the interventionalist. An overall threshold for training of generic cases, e.g. simulator hours, and beginning of specific rehearsal training according pre-interventional complexity grading and case selection could assist the ongoing development of training curricula with angiography simulators. Of note, multidisciplinary and interprofessional team interventions might not be solely evaluated using technical parameters but also communication skills, which can be evaluated with additional instruments [31]. Third, the design of future studies investigating the training effect on angiography simulators should be focussed on randomized trials and include outcome parameter with clinical impact. Although the selection of clinically relevant outcome parameters is dependent on the specific intervention, technical success and procedure-related complications would be of general interest if standardized reported. Large cohort or registry studies would support to evaluate the training effect of angiography simulators on clinical work and benefit for the patient.

## Conclusion

Applying angiography simulators in interventional training can reduce PT in real patient interventions. The effect on FT is comparable between training with and without AST. Angiography simulators offer a valuable, radiation-free alternative compared to conventional training and expand training opportunities. Evidence of the findings is limited by study heterogeneity.

## Supplementary Information

Additional file 1: Sim additional file supplemental methods final.

Additional file 2: Sim additional file supplemental results final.

## Data Availability

Initial unpublished data were part of a review by J.M.D. and T.C.M. for the scientific module in medical school. All data generated or analysed during this study are included in this published article and its supplementary information files.

## Abbreviations

AST: Angiography simulator training
CA: Carotid angiography
CIRSE: Cardiovascular and Interventional Society of Europe
EVAR: Endovascular aortic repair
FT: Fluoroscopy time
NA: Neurovascular angiography
n.a: Not available
PRISMA: Preferred Items for Systematic Review and Meta-Analysis
PT: Procedure time
SCAI: Society of cardiovascular angiography and interventions
USA: United States of America
VIST: Vascular interventional simulation trainer
