# Supplementary material for: Comparison of fluoroscopy time and procedure time of endovascular interventions with and without prior angiography simulator training: a meta-analysis
Source: Adv Simul (Lond). 2025 Oct 27;10:53. doi: 10.1186/s41077-025-00382-y (PMC12560282; doi:10.1186/s41077-025-00382-y)
Supplement: Supplementary file 1 — Additional file 1: Sim additional file supplemental methods final. [file 41077_2025_382_MOESM1_ESM.docx]

**Systematic literature research**

PubMed: 1653 records

((angiography simulator) OR (endovascular simulator)) AND ((fluoroscopy time) OR (procedure time))

WOS: 109 records

((ALL=angiography simulator) OR (ALL=endovascular simulator)) AND ((ALL=fluoroscopy time) OR (ALL=procedure time))

CINAHL: 2 records

(angiography simulator OR endovascular simulator) AND (fluoroscopy time OR procedure time)

**Study quality**

**Table 4. Study quality for procedure time**

| **Procedure time/ Study** | **Cates et al. (2016)** | **Desender et al. (2016)** | **Jensen et al. (2014)** | **Jensen et al. (2016)** | **Kreiser et al. (2020) A** | **Kreiser et al. (2020) B** | **Popovic et al. (2019)** | **Prenner et al. (2017)** | **Våpenstad et al. (2021)** | **Wooster et al. (2018)** |
| --- | --- | --- | --- | --- | --- | --- | --- | --- | --- | --- |
| **Modified Downs-and-Black Checklist** |  |  |  |  |  |  |  |  |  |  |
| Is the hypothesis/aim/objective of the study clearly described? (Yes=1,No=0) | 1 | 1 | n.a. | 1 | n.a. | 1 | 1 | 1 | 1 | 1 |
| Are the main outcomes to be measured clearly described in the Introduction or Methods section? (Yes=1,No=0) | 1 | 1 | n.a. | 1 | n.a. | 1 | 1 | 1 | 1 | 1 |
| Are the characteristics of the patients included in the study clearly described? (Yes=1,No=0) | 1 | 1 | n.a. | 1 | n.a. | 1 | 1 | 1 | 1 | 1 |
| Are the main findings of the study clearly described? (Yes=1,No=0) | 1 | 1 | n.a. | 1 | n.a. | 1 | 1 | 1 | 1 | 1 |
| Were the subjects asked to participate in the study representative of the entire population from which they were recruited? (Yes=1,No=0) | 1 | 1 | n.a. | 1 | n.a. | 1 | 1 | 1 | 1 | 1 |
| Were those subjects who were prepared to participate representative of the entire population from which they were recruited? (Yes=1,No=0) | 1 | 1 | n.a. | 1 | n.a. | 1 | 1 | 1 | 1 | 1 |
| Were the statistical tests used to assess the main outcomes appropriate? (Yes=1,No=0) | 1 | 1 | n.a. | 1 | n.a. | 1 | 1 | 1 | 1 | 0 |
| Were the main outcome measures used accurate (valid and reliable)? (Yes=1,No=0) | 1 | 1 | n.a. | 1 | n.a. | 1 | 1 | 1 | 1 | 1 |
| Sum | 8 | 8 | n.a. | 8 | n.a. | 8 | 8 | 8 | 8 | 7 |

*In Table 4, the consensus of study quality assessment for procedure time was tabulated. Study quality was assessed using Downs-and-Black instrument modified by Zadro et al.[1]. Abbreviations: n.a. = not available.*

**Table 5. Study quality for fluoroscopy time**

| **Fluoroscopy time/ Study** | **Cates et al. (2016)** | **Desender et al. (2016)** | **Jensen et al. (2014)** | **Jensen et al. (2016)** | **Kreiser et al. (2020) A** | **Kreiser et al. (2020) B** | **Popovic et al. (2019)** | **Prenner et al. (2017)** | **Våpenstad et al. (2021)** | **Wooster et al. (2018)** |
| --- | --- | --- | --- | --- | --- | --- | --- | --- | --- | --- |
| **Modified Downs-and-Black Checklist** |  |  |  |  |  |  |  |  |  |  |
| Is the hypothesis/aim/objective of the study clearly described? (Yes=1,No=0) | 1 | 1 | 1 | 1 | 1 | 1 | 1 | 1 | 1 | 1 |
| Are the main outcomes to be measured clearly described in the Introduction or Methods section? (Yes=1,No=0) | 1 | 1 | 1 | 1 | 1 | 1 | 1 | 1 | 1 | 1 |
| Are the characteristics of the patients included in the study clearly described? (Yes=1,No=0) | 1 | 1 | 1 | 1 | 1 | 1 | 1 | 1 | 1 | 1 |
| Are the main findings of the study clearly described? (Yes=1,No=0) | 1 | 1 | 1 | 1 | 1 | 1 | 1 | 1 | 1 | 1 |
| Were the subjects asked to participate in the study representative of the entire population from which they were recruited? (Yes=1,No=0) | 1 | 1 | 1 | 1 | 1 | 1 | 1 | 1 | 1 | 1 |
| Were those subjects who were prepared to participate representative of the entire population from which they were recruited? (Yes=1,No=0) | 1 | 1 | 1 | 1 | 1 | 1 | 1 | 1 | 1 | 1 |
| Were the statistical tests used to assess the main outcomes appropriate? (Yes=1,No=0) | 1 | 1 | 1 | 1 | 1 | 1 | 1 | 1 | 0 | 1 |
| Were the main outcome measures used accurate (valid and reliable)? (Yes=1,No=0) | 1 | 1 | 1 | 1 | 1 | 1 | 1 | 1 | 1 | 1 |
| Sum | 8 | 8 | 8 | 8 | 8 | 8 | 8 | 8 | 7 | 8 |

*In Table 5, the consensus of study quality assessment for fluoroscopy time was tabulated. Study quality was assessed using Downs-and-Black instrument modified by Zadro et al.[1].*

**Data extraction**

**Table 6. Conversion of the reported values for procedure time**

| **Study** | **AST-group** | | **Control-group** | |
| --- | --- | --- | --- | --- |
|  | **n** | **values** | **n** | **values** |
| **Cates et al. (2016)** | 6 | CI: 18.13 min - 35.67 min | 6 | CI: 24.47 min - 40.21 min |
|  |  | *mean: 26.9 min^e^*  *SD: 8.3 min^f^* |  | *mean: 32.3 min^e^*  *SD: 7.4 min^f^* |
| **Desender et al. (2016)** | 50 | GM: 52.1 min  95% CI: 46.2 min – 58.8 min | 50 | GM: 54.6 min  95% CI: 48.4 min – 61.6 min |
|  |  | *mean: 57.0 min^g^*  *SD: 10.2 min^g^* |  | *mean: 59.7 min^g^*  *SD: 10.7 min^g^* |
| **Jensen et al. (2014)** | n.a. | n.a. | n.a. | n.a. |
| **Jensen et al. (2016)** | 16 | Q1: 1171 sec  median: 1356 sec  Q3: 1607 sec | 16 | Q1: 1401 sec  median: 1623 sec  Q3: 1890 sec |
|  |  | *mean: 1378 sec^a^ = 22.9 min*  *SD: 354.39 sec^a^ = 5.9 min* |  | *mean: 1638 sec^a^ = 27.3 min*  *SD: 397.46 sec^a^ = 6.6 min* |
| **Kreiser et al. (2020) A** | n.a. | n.a. | n.a. | n.a. |
| **Kreiser et al. (2020) B** | 20 | median: 32.5 min  IQR: 27.25 min | 20 | median: 30.0 min  IQR: 19.5 min |
|  |  | *mean: 32.5 min^b^*  *SD: 20.1 min^c^* |  | *mean: 30,0 min^b^*  *SD: 14.4 min^c^* |
| **Popovic et al. (2019)** | 40 | Q1: 10 min  median: 13 min  Q3: 18 min | 40 | Q1: 14 min  median: 16 min  Q3: 18 min |
|  |  | *mean: 13.6 min^a^*  *SD: 6.1 min^a^* |  | *mean: 16.0 min^a^*  *SD: 3.0 min^a^* |
| **Prenner et al. (2017)** | 895 | mean: 23.98 min  SD: 11.89 min | 1888 | mean: 24.94 min  SD: 10.88 min |
|  |  | *mean: 23.9 min*  *SD: 11.8 min* |  | *mean: 24.9 min*  *SD: 10.8 min* |
| **Våpenstad et al. (2021)** | 30 | minimum: 23 min  median: 44 min  maximum: 129 min | 30 | minimum: 27 min  median: 55 min  maximum: 132 min |
|  |  | *mean: 60.0 min^a^*  *SD: 25.9 min^a^* |  | *mean: 67.2 min^a^*  *SD: 25.7 min^a^* |
| **Wooster et al. (2018)** | 6 | minimum: 14.2 min  median: 31.9 min  maximum: 54 min | 9 | minimum: 30.9 min  median: 42.5 min  maximum: 69 min |
|  |  | *mean: 31.9 min^b^*  *SD: 15.7 min^d^* |  | *mean: 42.5 min^b^*  *SD: 12.8 min^d^* |

*In Table 6, the reported values for the procedure time were tabulated. Reported values were written in normal font and converted values were written in italic font. Values were truncated after first decimal position. Abbreviations: AST = angiography simulator training, CI = confidence interval, GM = geometric mean, IQR = interquartile range, min = minutes, n = number, n.a. = not available, Q1 = first quartile, Q3 = third quartile, SD = standard deviation, sec = seconds.*

*^a^Conversion according Wan et al.[2]*

*^b^Conversion according Cochrane Handbook [3]: mean = median*

*^c^Conversion according Cochrane Handbook [2,4]: SD = IQR/1.35*

*^d^Conversion according Walter et al. [5]: SD = range x f; f = 0.395 for n = 6*

*^e^Conversion according LibreTexts Statistics Library [6]: mean = (upper value + lower value) / 2*

*^f^Conversion according Cochrane Handbook [7]: SD = ((n^(1/2)) x (upper value - lower value) / (2 x (TINV(1-0.95,n-1))*

*^g^Conversion according Taylor and Higgins et al. [8,9]:*

- *mean = (exp((ln(GM)) + ((((((ln(upper value))-(ln(lower value))) x (n^(1/2)))/ (2 x (TINV(1-0.95,n-1))))^2^)/2)))*
- *SD = (((exp((((((ln(upper value))-(ln(lower value))) x (n^(1/2)))/ (2 x (TINV(1-0.95,n-1))))^2^))-1) x exp((2 x (ln(GM)))+(( ((((ln(upper value))-(ln(lower value))) x (n^(1/2)))/ (2 x (TINV(1-0.95,n-1))))^2^))))^(1/2)*

**Table 7. Conversion of the reported values for fluoroscopy time**

| **Study** | **AST-group** | | **Control-group** | |
| --- | --- | --- | --- | --- |
|  | **n** | **values** | **n** | **values** |
| **Cates et al. (2016)** | 6 | CI: 10.82 min - 16.48 min | 6 | CI: 12.58 min - 22.13 min |
|  |  | *mean: 13.6 min^e^*  *SD: 2.6 min^f^* |  | *mean: 17.3 min^e^*  *SD: 4.5 min^f^* |
| **Desender et al. (2016)** | 50 | GM: 916 sec  95% CI: 763 sec - 1099 sec | 50 | GM: 864 sec  95% CI: 720 sec – 1037 sec |
|  |  | *mean: 1125 sec^g^ = 18.7 min*  *SD: 467 sec^g^ = 7.7 min* |  | *mean: 1061 sec^g^ = 17.6 min*  *SD: 440 sec^g^ = 7.3 min* |
| **Jensen et al. (2014)** | 878 | median: 360 sec  IQR: 245 sec - 557 sec | 3594 | median: 289 sec  IQR: 179 sec - 468 sec |
|  |  | *mean: 387 sec^a^ = 6.4 min*  *SD: 231 sec^a^ = 3.8 min* |  | *mean: 312 sec^a^ = 5.2 min*  *SD: 214 sec^a^ = 3.5 min* |
| **Jensen et al. (2016)** | 16 | Q1: 494 sec  median: 558 sec  Q3: 609 sec | 16 | Q1: 710 sec  median: 842 sec  Q3: 962 sec |
|  |  | *mean: 553 sec^a^ = 9.2 min*  *SD: 93 sec^a^ = 1.5 min* |  | *mean: 838 sec^a^ = 13.9 min*  *SD: 204 sec^a^ = 3.4 min* |
| **Kreiser et al. (2020) A** | 90 | median: 7.25 min  IQR: 12 min | 90 | median: 11 min  IQR: 10 min |
|  |  | *mean: 7.2 min^b^*  *SD: 8.8 min^c^* |  | *mean: 11.0 min^b^*  *SD: 7.4 min^c^* |
| **Kreiser et al. (2020) B** | 20 | median: 13.06 min  IQR: 17.64 min | 20 | median: 8.16 min  IQR: 5.22 min |
|  |  | *mean: 13.0 min^b^*  *SD: 13.0 min^c^* |  | *mean: 8.1 min^b^*  *SD: 3.8 min^c^* |
| **Popovic et al. (2019)** | 40 | Q1: 6 min  median: 7 min  Q3: 8 min | 40 | Q1: 6 min  median: 8 min  Q3: 9 min |
|  |  | *mean: 7.0 min^a^*  *SD: 1.5 min^a^* |  | *mean: 7.6 min^a^*  *SD: 2.3 min^a^* |
| **Prenner et al. (2017)** | 895 | Q1: 186 sec  median: 270 sec  Q3: 430.80 sec | 1888 | Q1: 198 sec  median: 276 sec  Q3: 423.6 sec |
|  |  | *mean: 295 sec^a^ = 4.9 min*  *SD: 181 sec^a^ = 3.0 min* |  | *mean: 299 sec^a^ = 4.9 min*  *SD: 167 sec^a^ = 2.7 min* |
| **Våpenstad et al. (2021)** | 30 | minimum: 14 min  median: 24 min  maximum: 53 min | 30 | minimum: 14 min  median: 27 min  maximum: 55 min |
|  |  | *mean: 28.7 min^a^*  *SD: 9.5 min^a^* |  | *mean: 30.7 min^a^*  *SD: 10.0 min^a^* |
| **Wooster et al. (2018)** | 6 | minimum: 5.4 min  median: 11.4 min  maximum: 19.8 min | 9 | minimum: 9.8 min  median: 19.4 min  maximum: 38 min |
|  |  | *mean: 11.4 min^b^*  *SD: 5.6 min^d^* |  | *mean: 19.4 min^b^*  *SD: 9.5 min^d^* |

*In Table 7, the reported values for the fluoroscopy time were tabulated. Abbreviations: AST = angiography simulator training, CI = confidence interval, GM = geometric mean, IQR = interquartile range, min = minutes, n = number, n.a. = not available, Q1 = first quartile, Q3 = third quartile, SD = standard deviation, sec = seconds.*

*^a^Conversion according Wan et al.[2]*

*^b^Conversion according Cochrane Handbook [3]: mean = median*

*^c^Conversion according Cochrane Handbook [2,4]: SD = IQR/1.35*

*^d^Conversion according Walter et al. [5]: SD = range x f; f = 0.395 for n = 6*

*^e^Conversion according LibreTexts Statistics Library [6]: mean = (upper value + lower value) / 2*

*^f^Conversion according Cochrane Handbook [7]: SD = ((n^(1/2)) x (upper value - lower value) / (2 x (TINV(1-0.95,n-1))*

*^g^Conversion according Taylor and Higgins et al. [8,9]:*

- *mean = (exp((ln(GM)) + ((((((ln(upper value))-(ln(lower value))) x (n^(1/2)))/ (2 x (TINV(1-0.95,n-1))))^2^)/2)))*
- *SD = (((exp((((((ln(upper value))-(ln(lower value))) x (n^(1/2)))/ (2 x (TINV(1-0.95,n-1))))^2^))-1) x exp((2 x (ln(GM)))+(( ((((ln(upper value))-(ln(lower value))) x (n^(1/2)))/ (2 x (TINV(1-0.95,n-1))))^2^))))^(1/2)*

**References**

1. Zadro J, O’Keeffe M, Maher C. Do physical therapists follow evidence-based guidelines when managing musculoskeletal conditions? Systematic review. BMJ Open. 2019;9:e032329.

2. Wan X, Wang W, Liu J, Tong T. Estimating the sample mean and standard deviation from the sample size, median, range and/or interquartile range. BMC Med Res Methodol. 2014;14:135.

3. Cochrane Handbook. 7.7.3.5 Medians and interquartile ranges [Internet]. 2025. Available from: https://handbook-5-1.cochrane.org/chapter_7/7_7_3_5_mediansand_interquartile_ranges.htm. Accessed on 11 April 2025 at 9:00 pm.

4. Cochrane Handbook. 6.5.2.5 Interquartile ranges [Internet]. 2025. Available from: https://training.cochrane.org/handbook/current/chapter-06#section-6-5-2-5. Accessed on 11 April 2025 at 9:05 pm.

5. Walter SD, Yao X. Effect sizes can be calculated for studies reporting ranges for outcome variables in systematic reviews. J Clin Epidemiol. 2007;60:849–52.

6. LibreTexts Statistics Library. 8.2: A Single Population Mean using the Normal Distribution [Internet]. 2025. Available from: https://stats.libretexts.org/Courses/Los_Angeles_City_College/Introductory_Statistics/08%3A_Confidence_Intervals/8.02%3A_A_Single_Population_Mean_using_the_Normal_Distribution. Accessed on 11 April 2025 at 9:15 pm.

7. Cochrane Handbook. 7.7.3.2 Obtaining standard deviations from standard errors and confidence intervals for group means [Internet]. 2025. Available from: https://handbook-5-1.cochrane.org/chapter_7/7_7_3_2_obtaining_standard_deviations_from_standard_errors_and.htm. Accessed on 11 April 2025 at 9:10 pm.

8. Taylor K. Data Extraction for Meta-analysis - 25 [Internet]. 2025. Available from: google.de/url?sa=t&rct=j&q=&esrc=s&source=web&cd=&ved=2ahUKEwis77KplM2MAxWg-QIHHcU5EgYQFnoECBgQAQ&url=https%3A%2F%2Fwww.cebm.ox.ac.uk%2Ffiles%2Fdata-extraction-tips%2Fblog25revised_dataextractiontips_kathytaylor.pdf&usg=AOvVaw0BmS_mJ9p-54dxFNCIJRhj&opi=89978449. Accessed on 11 April 2025 at 9:15 pm.

9. Higgins JPT, White IR, Anzures‐Cabrera J. Meta‐analysis of skewed data: Combining results reported on log‐transformed or raw scales. Stat Med. 2008;27:6072–92.
