# Supplementary material for: Comparison of fluoroscopy time and procedure time of endovascular interventions with and without prior angiography simulator training: a meta-analysis
Source: Adv Simul (Lond). 2025 Oct 27;10:53. doi: 10.1186/s41077-025-00382-y (PMC12560282; doi:10.1186/s41077-025-00382-y)
Supplement: Supplementary file 2 — Additional file 2: Sim additional file supplemental results final. [file 41077_2025_382_MOESM2_ESM.docx]

**Subgroup analysis**

**Table 8. Subgroup analysis**

| **Study** | **Training type** | **Intervention type** | **Interventional experience*** | **Study size**° | **Simulator type** | **Study design** | **Subgroup analysis** | |
| --- | --- | --- | --- | --- | --- | --- | --- | --- |
| **Cates et al. (2016)** | generic | NA | high | </=30 | VIST | randomized | PT | FT |
| **Desender et al. (2016)** | specific | EVAR | high | >30 | AM | randomized | PT | FT |
| **Jensen et al. (2014)** | generic | CA | low | >30 | VIST | retrospective | n.a. | FT |
| **Jensen et al. (2016)** | generic | CA | low | </=30 | VIST | randomized | PT | FT |
| **Kreiser et al. (2020) A** | generic | NA | low | >30 | VIST | retrospective | n.a. | FT |
| **Kreiser et al. (2020) B** | specific | NA | high | </=30 | VIST | randomized | PT | FT |
| **Popovic et al. (2019)** | generic | CA | low | >30 | AM | randomized | PT | FT |
| **Prenner et al. (2017)** | generic | CA | high | >30 | VIST | retrospective | PT | FT |
| **Våpenstad et al. (2021)** | specific | EVAR | high | </=30 | VIST | retrospective | PT | FT |
| **Wooster et al. (2018)** | specific | NA | high | </=30 | AM | randomized | PT | FT |

*In Table 8, groups for subgroup analysis on training type, intervention type, interventional experience, study size and simulator type were tabulated for procedure time. Abbreviations: AM = Angio Mentor, CA = coronary angiography, EVAR = endovascular aortic repair, NA = neurovascular angiography, PT = procedure time, FT = fluoroscopy time, VIST = vascular interventional simulation trainer.*

**Interventional experience was defined as high, if consultants performed/ finished the intervention, and it was defined as low, if residents performed the intervention.*

*°Subgroups were divided by number of interventions </=30 and >30 interventions.*

**Table 9. Subgroup analysis of the procedure time**

| **Subgroup** | | **n** | **I^2^** | **MD** | **95% CI** | **p-value** |
| --- | --- | --- | --- | --- | --- | --- |
| **Training type** | **generic** | 4 datasets | 48% | -2.08 min | -4.52 min – 0.36 min | 0.07 |
|  | **specific** | 4 datasets | 32.5% | -3.29 min | -10.57 min – 4.00 min | 0.25 |
| **Intervention type** | **CA** | 3 datasets | 54.2% | -1.92 min | -5.44 min – 1.61 min | 0.14 |
|  | **EVAR** | 2 datasets | 6.7% | -3.23 min | -21.61 min – 15.15 min | 0.27 |
|  | **NA** | 3 datasets | 35.2% | -3.80 min | -18.99 min – 11.40 min | 0.39 |
| **Interventional experience** | **High*** | 6 datasets | 49.2% | -2.51 min | -5.86 min – 0.84 min | 0.11 |
|  | **Low*** | 2 datasets | 14.1% | -2.87 min | -13.63 min – 7.90 min | 0.18 |
| **Study size** | **</=30°** | 5 datasets | 25.6% | -4.41 min | -9.09 min – 0.28 min | 0.06 |
|  | **>30°** | 3 datasets | 21.5% | -1.47 min | -3.56 min – 0.63 min | 0.09 |
| **Simulator type** | **AngioMentor** | 3 datasets | 52.7% | -3.00 min | -8.82 min – 2.82 min | 0.16 |
|  | **VIST** | 5 datasets | 41.6% | -2.42 min | -5.79 min – 0.96 min | 0.12 |
| **Study design** | **randomized** | 6 datasets | 45.1% | -3.16 min | -5.73 min – -0.59 min | 0.03 |
|  | **retrospective** | 2 datasets | 20.6% | -1.66 min | -26.01 min – 22.68 min | 0.54 |

*In Table 9, subgroup analysis on training type, intervention type, interventional experience, study size and simulator type were tabulated for procedure time. Abbreviations: CA = coronary angiography, CI = confidence interval, EVAR = endovascular aortic repair, I^2^ = I-squared, MD = mean difference, n = number, NA = neurovascular angiography, VIST = vascular interventional simulation trainer.*

**Interventional experience was defined as high, if consultants performed/ finished the intervention, and it was defined as low, if residents performed the intervention.*

*°Subgroups were divided by number of interventions </=30 and >30 interventions.*

**Table 10. Subgroup analysis of the fluoroscopy time**

| **Subgroup** | | **n** | **I^2^** | **MD** | **95% CI** | **p-value** |
| --- | --- | --- | --- | --- | --- | --- |
| **Training type** | **generic** | 6 datasets | 98.6% | -1.62 min | -4.20 min – 0.95 min | 0.17 |
|  | **specific** | 4 datasets | 75.2% | -0.58 min | -8.61 min – 7.46 min | 0.83 |
| **Intervention type** | **CA** | 4 datasets | 99.3% | -0.91 min | -4.88 min – 3.06 min | 0.52 |
|  | **EVAR** | 2 datasets | 28.6% | 0.08 min | -18.45 min – 18.60 min | 0.97 |
|  | **NA** | 4 datasets | 79.4% | -2.58 min | -10.61 min – 5.45 min | 0.38 |
| **Interventional experience** | **High*** | 6 datasets | 82.3% | -0.90 min | -4.92 min – 3.12 min | 0.59 |
|  | **Low*** | 4 datasets | 96.8% | -1.82 min | -6.22 min – 2.58 min | 0.28 |
| **Study size** | **</=30°** | 5 datasets | 76.3% | -2.72 min | -8.18 min – 2.73 min | 0.24 |
|  | **>30°** | 5 datasets | 98.1% | -0.31 min | -2.64 min – 2.02 min | 0.73 |
| **Simulator type** | **AngioMentor** | 3 datasets | 86.8% | -1.49 min | -11.83 min – 8.84 min | 0.60 |
|  | **VIST** | 7 datasets | 98.8% | -1.38 min | -4.28 min – 1.52 min | 0.29 |
| **Study design** | **randomized** | 6 datasets | 90.0% | -1.67 min | -5.97 min – 2.63 min | 0.36 |
|  | **retrospective** | 4 datasets | 98.7% | -0.75 min | -4.32 min – 2.82 min | 0.55 |

*In Table 10, subgroup analysis on training type, intervention type, interventional experience, study size and simulator type were tabulated for fluoroscopy time. Abbreviations: CA = coronary angiography, CI = confidence interval, EVAR = endovascular aortic repair, I^2^ = I-squared, MD = mean difference, n = number, NA = neurovascular angiography, VIST = vascular interventional simulation trainer.*

**Interventional experience was defined as high, if consultants performed/ finished the intervention, and it was defined as low, if residents performed the intervention.*

*°Subgroups were divided by number of interventions </=30 and >30 interventions.*

**Leave-one-out sensitivity analysis**

**Table 11.** **Leave-one-out sensitivity analysis of the procedure time**

| **Left-out** | **I^2^** | **MD** | **95% CI** | **p-value** |
| --- | --- | --- | --- | --- |
| **Cates et al. (2016)** | 67.3% | -2.51 min | -4.72 min – -0.29 min | 0.03 |
| **Desender et al. (2016)** | 66.1% | -2.71 min | -5.21 min – -0.21 min | 0.04 |
| **Jensen et al. (2014)** | n.a. | n.a. | n.a. | n.a. |
| **Jensen et al. (2016)** | 65.7% | -2.38 min | -4.72 min – -0.05 min | 0.05 |
| **Kreiser et al. (2020) A** | n.a. | n.a. | n.a. | n.a. |
| **Kreiser et al. (2020) B** | 53.6% | -2.65 min | -4.48 min – -0.81 min | 0.01 |
| **Popovic et al. (2019)** | 49.7% | -2.80 min | -5.41 min – -0.19 min | 0.04 |
| **Prenner et al. (2017)** | 39.5% | -3.32 min | -5.61 min – -1.02 min | 0.01 |
| **Våpenstad et al. (2021)** | 64.9% | -2.50 min | -4.58 min – -0.42 min | 0.03 |
| **Wooster et al. (2018)** | 51.0% | -2.30 min | -4.00 min – -0.61 min | 0.02 |

*In Table 11, leave-one-out sensitivity analysis on were tabulated for procedure time. Abbreviations: CI = confidence interval, I^2^ = I-squared, MD = mean difference, n.a. = not available.*

**Table 12. Leave-one-out sensitivity analysis of the fluoroscopy time**

| **Left-out** | **I^2^** | **MD** | **95% CI** | **p-value** |
| --- | --- | --- | --- | --- |
| **Cates et al. (2016)** | 98.8% | -1.11 min | -3.59 min – 1.37 min | 0.33 |
| **Desender et al. (2016)** | 98.7% | -1.62 min | -4.09 min – 0.86 min | 0.17 |
| **Jensen et al. (2014)** | 94.2% | -1.71 min | -4.18 min – 0.75 min | 0.15 |
| **Jensen et al. (2016)** | 98.6% | -0.86 min | -3.20 min – 1.47 min | 0.42 |
| **Kreiser et al. (2020) A** | 98.7% | -1.02 min | -3.50 min – 1.45 min | 0.37 |
| **Kreiser et al. (2020) B** | 98.1% | -1.65 min | -3.71 min – 0.42 min | 0.10 |
| **Popovic et al. (2019)** | 98.7% | -1.45 min | -4.07 min – 1.16 min | 0.24 |
| **Prenner et al. (2017)** | 94.5% | -1.54 min | -4.13 min – 1.05 min | 0.21 |
| **Våpenstad et al. (2021)** | 98.9% | -1.29 min | -3.84 min – 1.27 min | 0.28 |
| **Wooster et al. (2018)** | 98.2% | -1.01 min | -3.13 min – 1.11 min | 0.30 |

*In Table 12, leave-one-out sensitivity analysis on were tabulated for fluoroscopy time. Abbreviations: CI = confidence interval, I^2^ = I-squared, MD = mean difference.*
